# Malnutrition: From Mother to Child

**DOI:** 10.4269/ajtmh.24-0012

**Published:** 2024-06-04

**Authors:** William A. Petri, Jennifer Hendrick, Rashidul Haque

**Affiliations:** ^1^ Department of Medicine, University of Virginia, Charlottesville, Virginia;; ^2^ icddr,b, Mohakhali, Dhaka, Bangladesh

## Abstract

Malnutrition in children, defined as a child who is more than 2 standard deviations below the norm for height- or weight-for-age, is a pervasive, population-wide problem in low- and middle-income countries. Malnutrition is associated with increased infant mortality and delayed neurocognitive development. A recent pooled analysis of more than 30 longitudinal cohort studies demonstrated that the conditions mothers lived in during pregnancy, and their access to adequate nutrition, was a major factor in the subsequent growth and health of their children. We review here this analysis and the hypothesis that interventions to address childhood malnutrition need to start before birth and continue throughout the critical first 1,000 days of life.

Important insights into the incidence and cause of child malnutrition have emerged from a pooled analysis of studies of children in the first 2 years of life from low- and lower-middle income countries (LMICs) in Africa, Asia, and Latin America.^1^^–^^3^ The analysis was conducted on a combined 33 longitudinal cohorts that studied 83,671 children with 662,763 measurements from birth to 2 years of age. The data were collected between 1987 and 2014 and included measurements of nutritional status on average every month from birth to 2 years of life.^1^^–^^3^ The new pooled analysis confirmed the tragic impact of malnutrition on child health. There was an 8-fold increased hazard of death before 24 months for children who suffered from a combination of severe wasting (weight-for-height less than 3 standard deviations below the norm; WHZ <–3) and stunting (height less than 2 standard deviations below the norm; HAZ<–2), albeit the mortality data was predominantly from two cohorts from Bangladesh and Zambia. Contrary to conventional wisdom, stunting alone was not associated with increased mortality.

Stunting was common, occurred early, and was irreversible in most children from these cohorts.^1^^,^^3^ In the pooled analysis, stunting was found to occur very early, within the first 6 months of life. In South Asia. 20% of children were stunted at birth, and 38% by 3 months of life. Importantly most children who became stunted remained stunted by 24 months of life, with a minority (5%–7%) having a reversal in stunting. However, even in children for whom stunting reversed, more than 20% ultimately suffering a relapse.^1^^,^^3^ Even children who were never stunted in the first 2 years of life had an average drop of 0.5 HAZ by 15 months of age. If one looks at the average across the population of length for age, by 2 years of life, 95% of LMIC children fell below average, indicative of a population-wide problem.

Wasting is more common than previously thought, with discovery of a much higher cumulative incidence than indicated by single time point prevalence measures in the past.^2^^,^^3^ At 2 years of age, only 5.6% of children were wasted; however, by this time point, 29% of children had suffered at least one episode of wasting since birth and 10% from two or more episodes. Wasting was seasonal in some cohorts, with the highest incidence during the rainy season, a time of food scarcity before the harvest. Wasting increased the risk of death before age 2 years by 2-fold. With the majority of first episodes of wasting occurring before 3 months of age, early episodes of wasting were more likely to be followed by wasting and stunting or prolonged wasting, indicating an especially vulnerable time nutritionally for children in the first months of life.^2^ Because this is a time that exclusive breastfeeding without complementary feeding is recommended, interventions for treatment or prevention of early wasting may need to be targeted at improving the opportunity for the mother to nurse and augmenting the nutritional status of the lactating mother.

Thus, of the variables measured, the mother’s height and the birth length of the child have the largest impact on child malnutrition. Short mothers were more likely to give birth to stunted children. Stunted children were in turn predestined to remain stunted through the first 2 years of life. Anticipated by earlier, single-site studies,^4^ these studies point to the first 1,000 days of life, from conception to age 2 years, as the optimal time to intervene.^2^^,^^3^^,^^5^ The illustration of stunting and wasting at birth in these cohorts further suggests a role for mother to child interventions in preventing malnutrition.^1^^–^^3^

To date, measures shown to be of limited effectiveness in the prevention or treatment of malnutrition include maternal nutritional supplementation during pregnancy^6^ and lipid-based complementary feeding from 6 to 24 months of age.^7^ In the future, we propose that a goal should be to understand the transgenerational contributions of microbiome, small intestinal function, and epigenetics to allow a multi-interventional approach to the prevention and treatment of malnutrition.^8^^,^^9^
